# Distinct clinical, neuroimaging and genetic profiles of late-onset cobalamin C defects (cb1C): a report of 16 Chinese cases

**DOI:** 10.1186/s13023-019-1058-9

**Published:** 2019-05-15

**Authors:** Xianling Wang, Yanhui Yang, Xuying Li, Cunjiang Li, Chaodong Wang

**Affiliations:** 1 Department of Neurology, Xuanwu Hospital, Capital Medical University, 45 Changchun Street, Xicheng District, Beijing, 100053 People’s Republic of China; 2 Department of Radiology, Xuanwu Hospital, Capital Medical University, Beijing, China; 3 Department of Neurobiology, Xuanwu Hospital, Capital Medical University, Beijing, China

**Keywords:** CblC disease, *MMAHC* gene, Neuroimaging, Phenotypic heterogeneity

## Abstract

**Objective:**

The importance of late-onset cobalamin C (cblC) disorder is underestimated in adults. Improved awareness on its clinical and neuroimaging features helps timely diagnosis and appropriate treatment.

**Methods:**

Totally 16 late-onset cblC cases were diagnosed based on clinical, biochemical findings and MMAHC gene mutation analysis. Clinical presentations, neuroimaging features and mutational spectrum were reviewed.

**Results:**

The case series included 10 males and 6 females, with average age of 22 (range 13–40) years. All the 16 patients displayed bilateral pyramidal tract signs, and most of the cases (13) had cognitive impairment. Other symptoms included psychiatric symptoms (6), epilepsy (6), peripheral nerve damage (5), ocular symptoms (4) and lower-limb thrombosis (1). The neuroimaging findings were dominated by cerebral atrophy (11/16), followed by white matter lesions (4), cerebellar lesions/atrophy (2) and spinal cord lesions (1). There were also 2 patients with normal imaging. All the *MMACHC* mutations were compound heterozygous, of which the most and second frequent was c.482G > A (p.R161Q; 15/16 case; allele frequency: 46.88%) and c.609G > A(p.W203X; 6/16 case; allele frequency: 18.75%). In addition, patients carrying frameshift mutations (deletion/duplication) presented more frequently with psychiatric symptoms (57.1%) and optic nerve damages (42.9%) than those carrying point mutations (22.2 and 11.1%, respectively). In contrast, peripheral nerve (44.4%) and white matter lesions (33.3%) were more frequently identified in point mutation- carriers. However, the differences did not achieve statistical significance (all *p* > 0.05).

**Conclusion:**

Compared to the early-onset form, late-onset cblC displayed some clinical, neuroimaging and mutational profiles, which warrants particular attention in adult neurologic practice. These findings not only broaden our insights into the genotypes and phenotypes of the disease, but highlight the importance of early diagnosis and initiation of appropriate treatments.

**Electronic supplementary material:**

The online version of this article (10.1186/s13023-019-1058-9) contains supplementary material, which is available to authorized users.

## Introduction

Methylmalonic academia (MMA) with homocysteinemia, cobalamin-C (cblC) type, is the most common subtype of defective intracellular cobalamin (vitamin B12) metabolism [1, 2]. cblC disease results from mutations in the MMACHC gene, which result in impaired conversion of dietary vitamin B12 or cobalamin (Cbl) to its two metabolically active forms, methylcobalamin (MeCbl) and adenosylcobalamin (AdoCbl). MeCbl and AdoCbl are essential coenzymes to methionine synthase and methylmalonyl-CoA mutase, whose functional deficiency leads to methylmalonic academia combined with homocysteinemia. Based on the age of onset, there are two distinct clinical subtypes of cblC disease, early-onset and late-onset [3]. The early-onset type presents in the neonatal and early infantile period with failure to thrive, acute neurological deterioration, macrocytic anemia, multisystem organ dysfunction, metabolic acidosis and visual impairment (retinopathy, optic atrophy), and has a poor prognosis even with early treatment [4, 5]. Late-onset cblC patients have been defined as the patients have overt symptoms after 4 years of age. Compared to the early-onset form, the late-onset cblC is less common and has less severe presentations and more favorable outcomes if treated promptly [1, 2]. However, the diagnosis of late-onset cblC disease was often delayed and missed in the adult neurology practice, due to the rarity of the disease and lack of awareness by adult neurologists [6].

In recent years, more and more late-onset cases have been diagnosed and the incidence of late-onset appears to be higher than the previous estimate. However, the sample sizes reported late-onset cblC cases in previous studies [6–8] were relatively small, and the total number of reported late-onset cblC cases is < 80 [6](calculated according to the latest literatures). In this study, 16 Chinese cases with late-onset cobalamin C disorder were diagnosed and confirmed by mutation analysis of the *MMACHC* gene (NM_015506.2). We aim to characterize the clinical and neuroimaging profiles, as well as the mutational spectrum and genotype-phenotype correlation of the late-onset cblC cases.

## Methods

All the 16 cases with late-onset Cobalamin-C (cblC) disease were identified in Department of Neurology of Xuanwu Hospital from April 2009 to June 2018. All these patients presented with acute or insidious onset and extensive neurological signs. Neuroimaging studies and routine lab tests (including blood and CSF) excluded the possibilities of common diseases such as vascular, neoplasmic, inflammatory, degenerative or demyelinating diseases. On the other hand, these features, together with the relatively young onset (compared to most of the adult-onset diseases), support the possibility of hereditary and/or metabolic diseases. In this view, a routine metabolic screening, including the measurement of C3 (propionylcarnitine), C3/C0 (free carnitine) and C3/C2 (acetylcarnitine) in the plasma by tandem mass spectrometry, the detection of methylmalonic acid in the urine by gas-chromatography mass spectrometry, and the determination of total levels of homocysteine, vitamin B12 and folate in the serum. All the identified cases showed a significant increase of methylmalonic acid in the urine and serum homocysteine but without decrease of vitamin B12, which are indicative of cblC. Thus, a subsequent genetic test were performed to screen mutations of MMACHC, the causative gene for cblC by polymerase chain reaction (PCR) and direct DNA sequencing, as described previously [9]. Brain MRI, electromyography (EMG), electroencephalogram (EEG), and funduscopy examination were performed in all cases, and spinal cord MRI was performed in 7 cases. After re-evaluation and diagnostic confirmation by two senior neurologists and a geneticist, all cases were treated with parenteral hydroxocobalamin combined with oral betaine, folate and carnitine for 3–4 weeks. The responses to the treatments were reported by the patients. Except case no.1, neuroimaging follow-up were not conducted for these cases after treatment.

Mutational spectrum of MMACHC gene, and clinical and imaging features were compared between patients carrying frameshift (deletion/duplication) and point mutations using chi-square or Fisher Exact Test.

## Results

### Clinical features and biochemical findings

The cases series included 10 males and 6 females. The average age was the 22 years (range from 13 to 40), and the average onset age was 19 years (range from 11 to 40). Time between the first symptom and diagnosis ranged from 1 month to more than 10 years. Case 9 and 10 were siblings. The other cases were not related. The onset was acute or insidious and the diet changes, pregnancy, fever were most common triggers for the acute onset. The clinical presentations involve multiple neurological systems (Table 1). Cognitive impairment and psychiatric symptoms were the most common symptoms, which were observed in 13 and 6 cases, respectively. Four cases had epilepsy and two had epileptic discharges in EEG without clinical episode. Physical examination revealed bilateral pyramidal tract signs in all cases. Four cases (No.5, 7, 11, 12) presented with progressive paraplegia and bilateral pyramidal tract signs without sensory dysfunction, which were initially misdiagnosed with hereditary spastic paraplegia. Peripheral nerve damaging was detected in 5 cases by neurological examinations and/or EMG examinations. Ocular symptoms were found in 4 cases, among which 2 cases complained blurred vision and 2 cases did not have optic symptoms but were identified to have mild optic nerve atrophy and pigmentary retinal dystrophy by funduscopy. The renal function and hematological condition of all patients were normal. Deep vein thrombosis in the lower extremities was found in one case. The urine metabolic screening detected remarkably elevated urine MMA level in all cases. Elevated plasma homocysteine level was found in all the cases (Table 2), but the level of vitamin B12 and folate in the serum were normal or mildly elevated. By treating with parenteral hydroxocobalamin combined with oral betaine, folate and carnitine for 3–4 weeks, the majority of patients showed marked decrease of the urine MMA and plasma homocysteine levels, as well as different extent of symptomatic improvement. The symptomatic improvement upon treatment has been detailed in Table 1. Among these, the improvement of cognitive decline and psychiatric symptoms were most evident.

**Table 1:** Clinical information of 16 cases with late-onset cblC disease

Case No.	Gender	Age (year)	Duration	Onset symptom	Psychiatric symptoms	Cognitive impairment	Epilepsy	Pyramidal tract impairment	Peripheral nerve damage (EMG findings)	Visual impairment other symptoms	Response to treatment
1	F	14	1 m	Cognitive impairment	Irritability psychosis	Moderate memory decline	–	Paraplegia Bilateral Pyramidal tract signs	Sensorimotor polyneuropathy of lower limbs (involved both axon and myelin)	–	Memory decline and psychiatric symptoms were remarkably recovered
2	M	14	8 m	Cognitive impairment	Apathy lethargy depression	Severe cognitive impairment deterioration in school performance	Epileptic discharges in EEG	Bilateral Pyramidal tract signs	–	–	Cognitive impairment was partly recovered
3	M	40	1 m	Cognitive impairment	–	Moderate memory impairment	–	Quadriplegia Bilateral Pyramidal tract signs	Sensorimotor polyneuropathy of lower limbs (involved both axon and myelin)	–	All symptoms were fully recovered
4	F	22	3 m	Cognitive impairment	–	Moderate impaired memory and calculation ability	–	Paraplegia Bilateral Pyramidal tract signs	Sensorimotor polyneuropathy of lower limbs (involved myelin)	–	Moderate improvement of cognitive impairment
5	F	18	7y	Gait disturbance	–	Mild memory impairment	Epileptic discharges in EEG	Progressive spastic paraplegia Bilateral Pyramidal tract signs	–	–	Mild improvement of gait disturbance
6	F	13	5 m	Cognitive impairment	Irritability, aggressiveness	Moderate cognitive impairment, deterioration in school performance	–	Mild paraplegia Bilateral Pyramidal tract signs	–	–	Remarkable improvement of cognitive impairment
7	M	26	4 m	Weakness of lower limbs	–	–	Generalized tonic-clonic seizures	Progressive spastic paraplegia, Bilateral Pyramidal tract signs	–	Visual acuity: normal, Funduscopy examination: optic nerve atrophy	Weakness of lower limbs was remarkably recovered
8	M	16	2 m	Weakness of lower limbs	–	–	Generalized tonic-clonic seizures	Paraplegia Bilateral Pyramidal tract signs	–	–	Weakness of lower limbs was remarkably recovered
9	M	32	1.5y	Psychiatric symptoms	Euphoria, agitation auditory and vision hallucinations aggressiveness	Mild memory impairment	–	Paraplegia Bilateral Pyramidal tract signs	–	Visual acuity: normal. Funduscopy examination: mild optic nerve atrophy pigmentary retinal dystrophy	Psychiatric symptoms were remarkably recovered
10	M	29	9 m	Psychiatric symptoms	Euphoria, agitation irritability aggressiveness	Mild memory impairment	–	Paraplegia Bilateral Pyramidal tract signs	–	–	Psychiatric symptoms were remarkably recovered
11	F	15	1y	Weakness of lower limbs	–	Mild memory impairment	–	Progressive spastic paraplegia Bilateral Pyramidal tract signs	–	–	Weakness of lower limbs was partly recovered
12	M	23	2 m	Weakness of lower limbs	–	Mild memory impairment		Progressive spastic paraplegia Bilateral Pyramidal tract signs	–	–	Weakness of lower limbs was remarkably recovered
13	M	15	4 m	Cognitive impairment	–	Impaired memory and calculation ability		Mild paraplegia Bilateral Pyramidal tract signs	Sensory polyneuropathy of lower limbs (involved axon)	Decreased vision (right eye0.15, left eye 0.5, optic nerve damage)	Moderate improvement of vision and weakness
14	M	29	11y	Cognitive impairment	–	Moderate impaired memory speech difficulties	–	Paraplegia Bilateral Pyramidal tract signs	Sensorimotor polyneuropathy of four limbs (involved both axon and myelin)	–	Mild improvement of cognitive impairment
15	M	20	6y	Epilepsy	–	–	Generalized tonic-clonic seizures	Bilateral Pyramidal tract signs	–	Thrombosis of peroneal vein and intramuscular vein of left lower limb	Epilepsy and thrombosis were improved with antiepileptic and anticoagulant drugs
16	F	24	10y	Decreased vision depression	Moderate depression	Mild memory impairment	Generalized tonic-clonic seizures	Paraplegia Bilateral Pyramidal tract signs	–	Decreased vision(left eye 0.2 right eye 0.6 bilateral optic nerve atrophy)	Mild improvement of vision and depression

Bilateral Pyramidal tract signs presented positive Babinski sign in all cases on neurological examination

**Table 2:** Neuroimaging presentations, gene mutations, plasma homocysteinemia and urine MMA level of 16 cases with late-onset cblC disease

Case No.	Brain MRI	Spinal cord MRI	Plasma homocysteinemia level (μmol/L, normal range: 5.0–15.0)	MMA level (μg/mg creatinine, normal range: 0.2–3.6)	MMAHC mutation
1	Bilateral white matter lesions in the centrum ovale and corona radiata	Thoracic lesions	101.60	232.18	c.482G > A, c.609G > A
2	Bilateral white matter lesions in the bilateral periventricular white matter	–	135.7	191.22	c.482G > A, c.567dupT
3	Cerebral atrophy and bilateral cerebellar cortex lesions	–	57.2	70.53	c.482G > A, c.1A > G
4	Bilateral white matter lesions	–	79.8	166.64	c.482G > A, c.609G > A
5	Cerebral atrophy	Normal	99	321.12	c.482G > A, c.609G > A
6	Mild cerebral atrophy	–	88	340.8	c.482G > A, c.626dupT
7	Cerebral atrophy	Normal	97.7	172.4	c.567dupT, c.565C > A
8	Cerebral atrophy and white matter lesions in unilateral posterior ventricular area.	–	99.1	189.16	c.467G > A, c.482G > A
9	Normal	–	115.3	253.68	c.482G > A,c.656_658del
10	Mild cerebral atrophy	–	75.7	262.03	c.482G > A, c.656_658del
11	Cerebral atrophy	Normal	121	168.05	c.482G > A, c.427C > T
12	Normal	Normal	93.6	81.62	c.482G > A, c.609G > A
13	Cerebral atrophy and bilateral cerebellum atrophy	–	102.6	58.42	c.482G > A, c.658_660del
14	Cerebral atrophy	–	86	288.85	c.482G > A, c.609G > A
15	Hippocampus atrophy	Normal	114.1	116.04	c.326_329del, .482G > A
16	Mild cerebral atrophy	Normal	124.5	184.71	c.482G > A, c.609G > A

### Neuroimaging findings

**Fig. 1:**
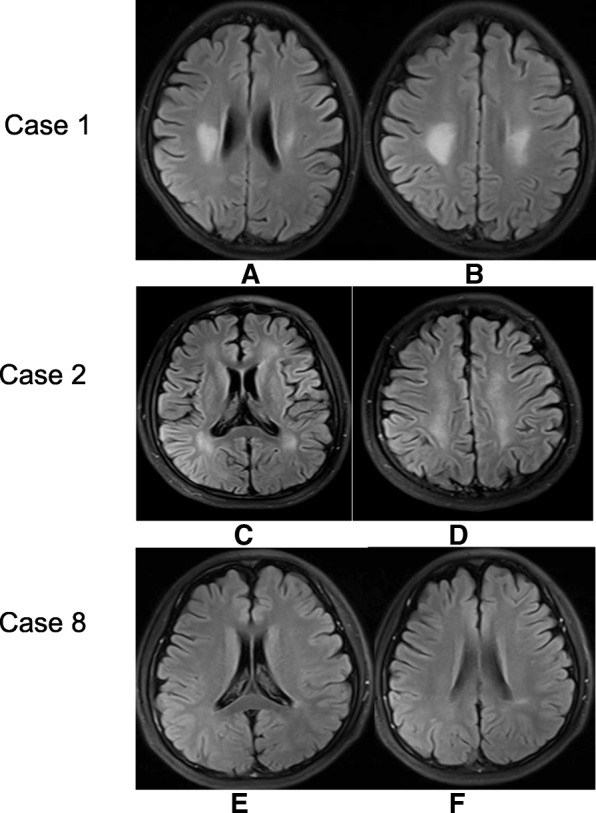
White matter lesions in three cases with late-onset cblC disease. The brain MRI of case 1 in Table 1 showed symmetric patchy lesions in corona radiata (**a**) and centrum ovale (**b**). The brain MRI of case 2 in Table 1 showed symmetric patchy lesions in bilateral periventricular white matter, especially in posterior area (**c**) and corona radiata (**d**). The MRI of case 8 in Table 1 showed the small lesions in unilateral posterior ventricular area (**e, f**). The white matter lesions in these three cases presented hyperintensity on fluid attenuated inversion recovery (FLAIR) image

**Fig. 2:**
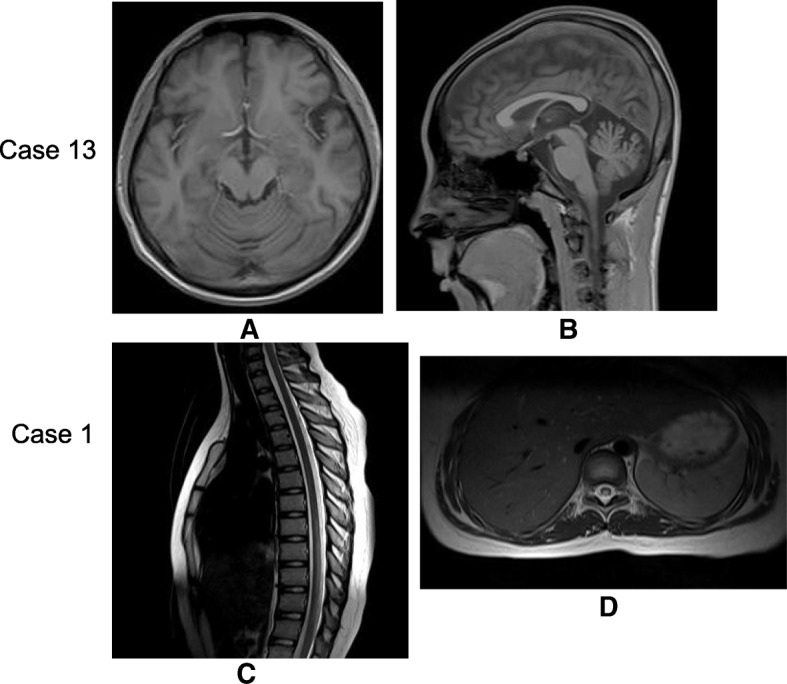
Cerebellum atrophy and the spinal cord lesions in late-onset cblC disease. The brain MRI of case 13 in Table 1 showed the cerebellum atrophy on T1 weighted image (**a**: transverse section, **b**: sagittal section). The spinal cord MRI of case 1 in Table 1 showed the spinal cord lesions in T8–11. The lesions presented hyperintensity on T2 weighted image (**c**: sagittal section, **d**: transverse section)

The neuroimaging findings were dominated by various degree cerebral atrophy (11/16), followed by high-intensity lesions in white matter (4/16), cerebellar lesions (1/16), cerebellar atrophy (1/16) and spinal cord lesions (1/16). There were also 2 patients with normal imaging (Table 2). Different from the diffuse white matter swelling in early-onset cblC cases, the white matter lesions in these late-onset cases presented with bilateral symmetric patchy lesions mainly in centrum ovale, corona radiate and periventricular area (Fig. 1a-d). Case 8 only presented small lesions in unilateral posterior ventricular area (Fig. 1e, f). Cerebellum lesions were rarely reported in cblC cases. In this study, bilateral cerebellar cortex lesions were found in one case (firstly reported in our previous report [8]) and cerebellum atrophy in case 13 (Fig. 2a, b). The spinal cord MRI examinations showed spinal cord lesions in case 1 (Fig. 2c, d) and the lesions disappeared after treatment. None of the cases showed the basal ganglion lesions, hydrocephalus or diffused white matter swelling, which were the common features in early-onset cobalamin C disorder. Only case No. 4 had MR spectroscopy data from areas of bilateral centrum ovale and corona radiata lesions, which showed decreased N-acetylaspartate (NAA) and increased choline (Cho) and lactate (Lac) peak in lesions of in both sides.

### Mutation and genotype-phenotype correlation analyses

**Fig. 3:**
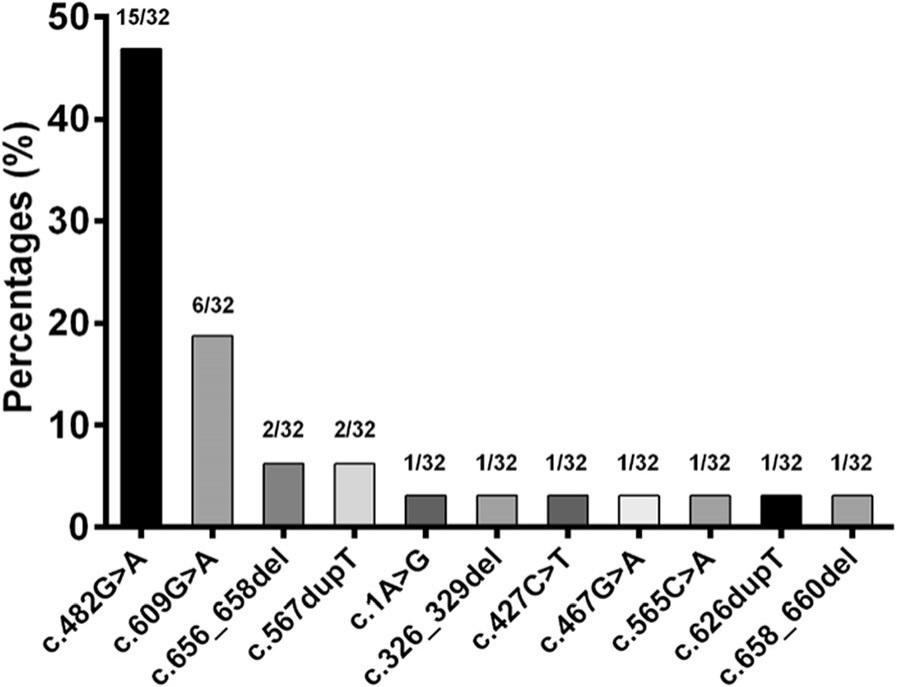
Spectrum of MMACHC gene mutations in late-onset cblC disease

*MMACHC* mutations were detected in all the 16 cases, including 11 Known mutations. All the mutations in *MMACHC* gene were compound heterozygous mutations and c.482G > A (p.R161Q) was the most frequent cblC mutation detected in15/16 patients, affecting 15 of 32 *MMACHC* alleles (46.88%). The second common mutation was c.609G > A(p.W203X) in 6/16 patients and affecting 6 of 32 *MMACHC* alleles (18.75%). c.567dupT(p.R189fs) and c.656_658del(p.219_220del) was respectively found in 2 /16 cases (Table 2). The spectrum of MMACHC gene mutations in these late-onset cases was shown in Fig. 3. The c.271dupA and c.331 C > T mutations, which was the most commonly related with early-onset form of the cblC defect [1, 2], was not found in these late-onset cases. c.394C > T mutation, which was most commonly related to late-onset disease in previous studies [1, 2], was not found in these late-onset cases. Comparing the allele frequencies for each detected mutation between our patient series and previously published studies and in the public database (ExAC, gnome AD), the c.482G > A variant in our cases were much more prevalent than other reported cases, and c.609G > A seemed to be ethnically related to Chinese patients (Additional file 1: Table S1).

Among all MMACHC mutations, frameshift and point mutation accounted for 56.3% (9/16) and 43.8% (7/16), respectively. And patients with symptoms involving damage from peripheral nerve and white matter accounted for 31.3% (5/16) and 25% (4/16), respectively. Comparing clinical and imaging features (Table 3), patients carrying frameshift mutations (deletion/duplication) presented more frequently with psychiatric symptoms (57.1%) and optic nerve damages (42.9%) than those carrying point mutations (22.2 and 11.1%, respectively). In contrast, peripheral nerve (44.4%) and white matter lesions (33.3%) were more frequently identified in point mutation-carriers. However, the differences did not achieve statistical significance (all *p* > 0.05).

**Table 3:** Comparison of the clinical symptoms and neuroimaging presentations between the cases with point mutations and frameshift mutations

	Point mutation (*n* = 9)	Frameshift mutation (*n* = 7)	χ^2^	p
psychiatric symptom (+)	2 (22.2)	4 (57.1)	2.05	0.15
psychiatric symptom (—)	7 (77.8)	3 (42.9)		
cognitive impairment(+)	8 (88.9)	5 (71.4)	0.79	0.38
cognitive impairment(—)	1 (11.1)	2 (28.6)		
epilepsy(+)	3 (33.3)	3 (42.9)	0.15	0.70
epilepsy(—)	6 (66.7)	4 (57.1)		
Peripheral nerve(+)	4 (44.4)	1 (14.3)	1.67	0.20
Peripheral nerve(—)	5 (55.6)	6 (85.7)		
optic nerve damage(+)	1 (11.1)	3 (42.9)	2.12	0.15
optic nerve damage(—)	8 (88.9)	4 (57.1)		
white matter lesions(+)	3 (33.3)	1 (14.3)	0.76	0.38
white matter lesions(—)	6 (66.7)	6 (85.7)		
cerebral atrophy(+)	6 (66.7)	5 (71.4)	0.04	0.84
cerebral atrophy(—)	3 (33.3)	2 (28.6)		
spinal cord lesions(+)	1 (11.1)	0 (0.0)	0.83	0.36
spinal cord lesions(—)	8 (88.9)	7 (100.0)		
cerebellum lesions(+)	1 (11.1)	1 (14.3)	0.04	0.85
cerebellum lesions(—)	8 (88.9)	6 (85.7)		

## Discussion

Using the largest series of Chinese cases, we comprehensively analyzed the clinical and imaging and genetic features of late-onset cblC. Compared with the early-onset type, the late-onset cblC presents more extensive but milder symptoms of the nervous system, and has a much better prognosis. Except one with thromboembolic complications, none of the cases had multisystem organ dysfunction and metabolic acidosis. In general, neuroimaging findings showed less severe damages in the central nervous system (CNS) among the late-onset patients. The cases displayed more cerebral atrophy and focal/patchy deep white matter lesions than early-onset ones without extensive and severe swelling and hydrocephalus. Moreover, the mutational spectrum of the *MMACHC* gene in late-onset cblC is significantly different from that in the early-onset type, as well as that previously reported in the late-onset cases.

The common presentations in these late-onset cblC cases include cognitive impairment, psychiatric symptoms, epilepsy, pyramidal tract signs and peripheral neuropathy, which is similar to a previous report [4, 10]. In addition, physical exam revealed frequent (100%) bilateral pyramidal tract signs but rare compromise in deep somatic sensory, which is different from subacute combined degeneration of the spinal cord (SCD), and easily misdiagnosed as hereditary spastic paraplegia (HSP). Spastic paraparesis is one of the multiple presentations of inborn errors of metabolism (IEMs) in children and adults, and even the only symptom for years in some cases. Therefore, it is essential not only to recognize the spastic paraparesis as one of the manifestations of IEMs, but also to include IEMs in the general diagnostic approach to spastic paraparesis [11]. Contrasting to previous reports, damage in optic and peripheral nerves was not rare, but the degree was relatively mild and even subclinical [12]. Moreover, the manifestations and severity vary among cases, suggesting considerable clinical heterogeneity of the diseases.

The neuroimaging features of late-onset cblC have not been clearly established. Diffuse supratentorial white matter swelling, variably severe white matter loss, hydrocephalus, thinning of the corpus callosum, symmetric bilateral lesions in the basal ganglia, the common and characteristic imaging findings in early-onset cblC [5, 13, 14], were not revealed in our late-onset cases. In contrast, cerebral atrophy and patchy lesions in deep white matter were common in late-, but not early-onset cases. In addition, we first identified 2 patients with high-intensity lesions (T2-weighted) or atrophy in the bilateral cerebellar hemisphere.

Over 75 *MMACHC* mutations have been detected in cblC diseases [1, 2, 14], among which c.271dupA and c.331C > T are the most common for early-onset cblC and c.394C > T was associated mainly with the late-onset subtype [13, 15–18]. However, none of them was detected in our late-onset cases. We revealed c.482G > A as the most common (15/16) cause of the late-onset cblC in Chinese, which is also highly prevalent in other ethnicities including those with Hispanic, Caucasian and mixed backgrounds [19, 20]. Homozygous c.609G > A is the second common mutation related to early-onset cb1C [21], but all mutations detected in our late-onset cases were heterozygotes. Moreover, patients carrying different mutations presented with distinct clinical and imaging features and showed different genotype-phenotype correlation from previous reports.

The molecular mechanisms underlying the phenotypic differences between the early- and late-onset cblC remain elusive, but may be related to the distinct mutational spectrum and different functional effects of these mutations. In general, late-onset cases rarely carry the homozygous mutations (especially nonsense and frameshift), which are functionally more pathogenic than heterozygous ones. At transcript level, different mutations have different levels of allelic expression and influence the *MMACHC* mRNA transcript level to different degrees. The early-onset c.271dupA mutation was underexpressed compared with the late-onset mutations and the *MMACHC* mRNA transcript levels in cell lines homozygous for the late-onset c.394C > T mutation had significantly higher transcript levels than those for the early-onset mutations [16]. At the protein level, pathogenic mutations, such as R161G and R161Q, can specifically impair the catalytic activities of MMACHC [22]. Moreover, other phenotypic modifiers, including the intracellular reactive oxygen species (ROS) and rate of apoptosis, may influence the expressivity and severity of different subtypes of cblC cases [23]. Nevertheless, all these evidences were obtained in cell models, and animal models are lacking for systemically investigating the molecular machineries underlying the phenotypic diversity.

## Conclusion

In summary, using one of the largest late-onset cblC case series, we have characterized the distinct clinical, neuroimaging and genetic profiles of the disease in Chinese. These findings not only broaden our insights into the genotypes and phenotypes of the disease, but also increase our awareness of these inborn errors of metabolisms in adult neurology practice to improve the diagnosis and appropriate treatment. Moreover, the unique mutational spectrum and genotype-phenotype correlation implied by these late-onset cases highlights the importance of early diagnosis and initiation of appropriate treatments.

## Additional file

Additional file 1: **Table S1.** Comparison of allele frequencies of identified mutations between our patients and other reports. (DOC 48 kb)

## Abbreviations

AdoCbl: Adenosylcobalamin
Cbl: Cobalamin
CblC: Cobalamin C disease
EEG: Electroencephalogram
EMG: Electromyography
FLAIR: Fluid attenuated inversion recovery
HSP: Hereditary spastic paraplegia
MeCbl: Methylcobalamin
MMA: Methylmalonic academia
PCR: Polymerase chain reaction
ROS: Reactive oxygen species
SCD: Subacute combined degeneration of the spinal cord
