# Supplementary material for: Distinct clinical, neuroimaging and genetic profiles of late-onset cobalamin C defects (cb1C): a report of 16 Chinese cases
Source: Orphanet J Rare Dis. 2019 May 15;14:109. doi: 10.1186/s13023-019-1058-9 (PMC6521494; doi:10.1186/s13023-019-1058-9)
Supplement: Supplementary file 1 — Table S1. Comparison of allele frequencies of identified mutations between our patients and other reports. (DOC 48 kb) [file 13023_2019_1058_MOESM1_ESM.doc]

**Supplementary materials**

**Table S1** Comparison of allele frequencies of identified mutations between our patients and other reports

| **Variant** | **Minor allele frequency (MAF)** | | | |
| --- | --- | --- | --- | --- |
|  | **Our cases** | **Previous reports** | **ExAC_**  **East Asian** | **gnomAD_**  **East Asian** |
| c.482G>A | 46.88% | 7%（Liu et al. (2010)） | 0.000149499 | 0.000149499 |
|  |  | [24% (Almannai et al, 2017)](https://www.ncbi.nlm.nih.gov/pubmed/?term=Almannai M%5BAuthor%5D&cauthor=true&cauthor_uid=28693988) |  |  |
|  |  | 4.37% (Hu S,2018) |  |  |
| c.609G>A | 18.75% | 48.1%（Liu et al. (2010)） | 3.31E-05 | 0.00004061 |
|  |  |  |  |  |
| c.656_658del | 6.25% | - | - | - |
|  |  |  |  |  |
| c.567dupT | 6.25% | 6.75% (Hu S,2018) | 3.23E-05 | 0.00003.23311 |
|  |  |  |  |  |
| c.1A>G | 3.13% | 2.2%（Liu et al. (2010)） | 8.28E-06 | 8.27993E-06 |
|  |  | 0.03%, (PMID:17768669) |  |  |
| c.326_329del | 3.13% | - | - | 0.00006095 |
|  |  |  |  |  |
| c.427C>T | 3.13% | - | - | - |
|  |  |  |  |  |
| c.467G>A | 3.13% | 0.79% (Hu S,2018) | - | - |
|  |  |  |  |  |
| c.565C>A | 3.13% | 11 % ( PMID:27289364) | 1.66E-05 | 1.65601E-05 |
|  |  |  |  |  |
| c.626dupT | 3.13% | 0.40% (Hu S,2018) | - | - |
|  |  |  |  |  |
| c.658_660del | 3.13% | 13.9%（Liu et al. (2010)） | - | 0.00004877 |
|  |  |  |  |  |

- Not reported
